# Primary intracranial malignant melanoma in an adolescent: case report and literature review

**DOI:** 10.3389/fsurg.2025.1524204

**Published:** 2025-02-10

**Authors:** Nyoman Golden, I Gusti Ketut Agung Surya Kencana, Christopher Lauren, Angky Saputra, Denny Japardi

**Affiliations:** Neurosurgery Division, Department of Surgery, Faculty of Medicine, Udayana University, Prof. Dr. I.G.N.G. Ngoerah General Hospital, Denpasar, Bali, Indonesia

**Keywords:** malignant melanoma, malignant neoplasm, neurosurgical procedures, oncology, tumor

## Abstract

Primary intracranial malignant melanoma (PIMM) is an exceedingly rare central nervous system tumor, accounting for only 1% of melanoma cases and 0.07% of primary CNS tumors, with limited documentation in adolescents. This case report describes an 18-year-old male who presented with a seizure, marking the onset of his symptoms. Following an emergency assessment, MRI identified a heterogeneous mass in the right parasagittal frontal region, initially misdiagnosed as a cystic meningioma. A craniotomy allowed for total tumor resection, and histopathological analysis revealed a malignant melanoma characterized by neoplastic cells with pronounced nuclear pleomorphism and significant mitotic activity. Postoperative evaluations, including a PET scan, confirmed no extracranial melanoma, affirming the diagnosis of primary CNS melanoma. The patient demonstrated no neurological deficits or seizures one year post-surgery and was managed with adjuvant radiotherapy. This report emphasizes the necessity of considering PIMM in differential diagnoses for seizures in young patients and highlights the importance of comprehensive diagnostic evaluations, including MRI and histopathology, in rare cases. Additionally, the findings underscore the critical role of complete surgical resection in improving outcomes, with adjuvant therapies potentially enhancing long-term management and surveillance. As PIMM presents with nonspecific symptoms, awareness among clinicians is essential for early detection and appropriate intervention, warranting further research to develop standardized treatment protocols and enhance understanding of this rare tumor's pathophysiology.

## 1 Introduction

Primary intracranial malignant melanoma (PIMM) is an extremely rare tumor of the central nervous system (CNS), with a very low incidence among all primary CNS tumors. PIMM accounts for only 1% of all melanoma cases and 0.07% of all primary CNS tumors (1–3). Most reported cases involve adults, with very few instances occurring in adolescents (2). The main purpose of this case report is to highlight a rare case of PIMM in an 18-year-old male, presenting with a seizure as the initial symptom. This report aims to contribute to the limited literature on PIMM in young patients and emphasize the clinical, radiological, and histopathological features that guided the diagnosis and management of this unusual condition.

## 2 Case description

### 2.1 History

An 18-year-old male patient presented to the emergency unit following a seizure that occurred two hours earlier. The seizure was the patient's first, characterized by upward eye deviation and generalized body convulsions. He was unconscious during the seizure, which lasted less than five minutes, and regained consciousness afterward. The patient also reported experiencing a generalized headache for the past two months, which temporarily improved with over-the-counter pain medications. There was no history of motor or sensory disturbances, nausea, or vomiting. The patient had no history of skin lesions or previous tumors. Additionally, there was no family history of similar symptoms or central nervous system or other systemic tumors.

### 2.2 Examination

**Figure 1:**
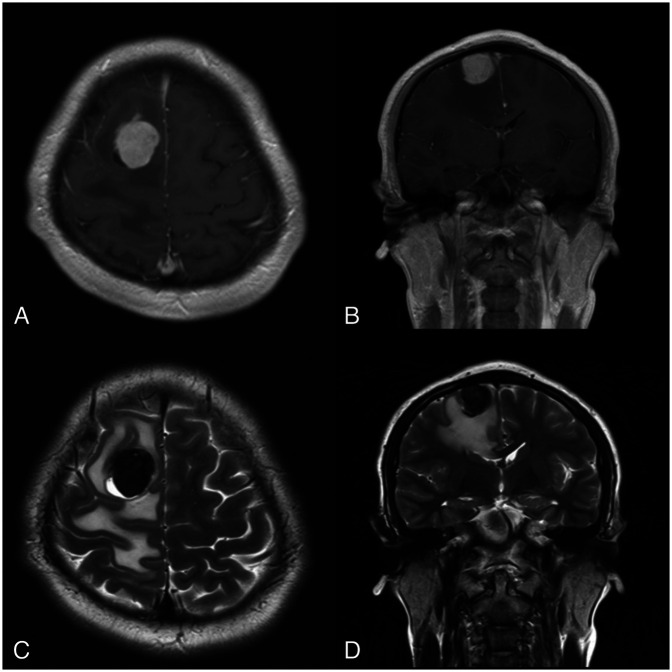
MRI reveals a focal mass located in the right parasagittal frontal region, surrounded by vasogenic edema. **(A,B)** Show the tumor mass enhancing with contrast on **(A)** axial and **(B)** coronal T1-weighted images. **(C,D)** Depict a cystic component within the tumor mass on **(C)** axial and **(D)** coronal T2-weighted images.

On physical examination, the patient had a Visual Analog Scale (VAS) score of 3/10 and a Karnofsky Performance Scale score of 90/100. General physical and neurological examinations were unremarkable. There were no visible skin lesions, hyperpigmentation, or similar abnormalities in the eye area or elsewhere on the body. MRI of the head revealed a heterogeneous solid extra-axial supratentorial mass in the right parasagittal frontal region, with cystic components and hemorrhage, accompanied by extensive surrounding vasogenic edema (Figures 1A–D). Based on these MRI findings, we provisionally diagnosed the patient with a parasagittal cystic meningioma, Type IV (according to Nauta's classification). The patient was scheduled for craniotomy and tumor removal.

### 2.3 Operative Procedure

**Figure 2:**
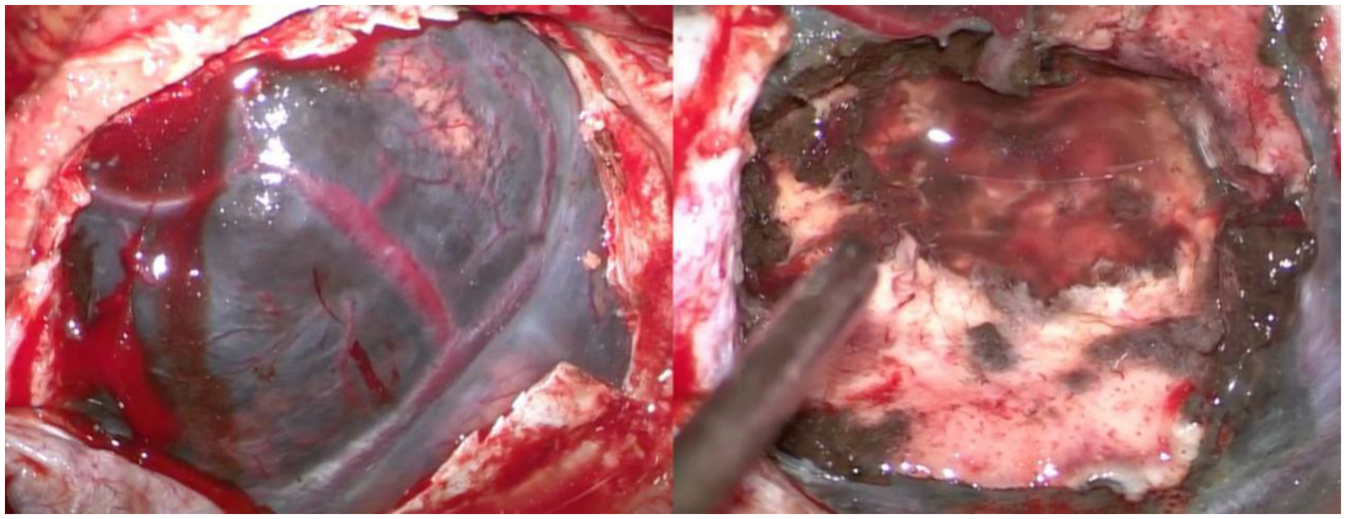
Intraoperative view. The dark-colored tumor mass is visible (left), and a subtotal tumor resection has been performed (right).

The patient was positioned supine with the head flexed. A layered skin incision was made, followed by a craniotomy. The dura mater was incised, revealing a dark gray mass (Figure 2). Intraoperative motor monitoring was not available, so the procedure was guided by anatomical landmarks, ensuring no disruption to the underlying cortex. A subtotal tumor resection was performed at that time because the tumor was firmly adherent to the cortical layer beneath it, and it was decided to leave behind the portion of the tumor that was tightly attached to the cortex. The resected tumor tissue was sent for histopathological examination. Hemostasis was achieved, and the wound was closed in layers.

### 2.4 Histopathological Findings

**Figure 3:**
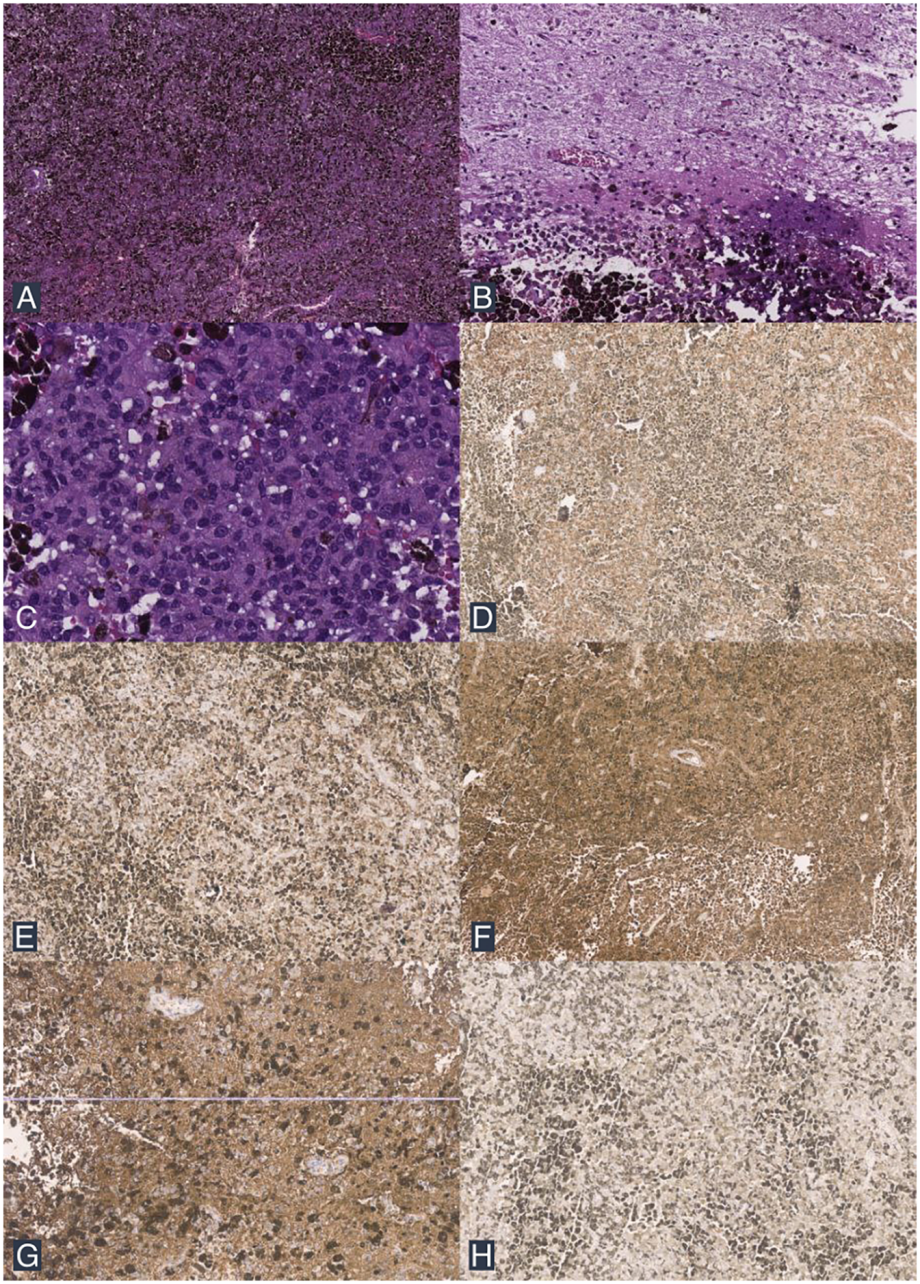
Histopathological findings. **(A)** The tumor mass consists of a diffuse proliferation of neoplastic cells with dense melanin pigment deposition (H&E stain; 40× magnification). **(B)** Infiltrative tumor cells are seen among the brain tissue (H&E stain; 100× magnification). **(C)** The tumor cells exhibit round to oval, polygonal to spindle morphology, with partially extensive eosinophilic cytoplasm, an increased N/C ratio, and pleomorphic nuclei (H&E stain; 200× magnification). **(D)** IHC shows positive staining for Melan A. **(E)** IHC shows positive staining for HMB45. **(F)** IHC shows positive staining for S100. **(G)** IHC shows positive staining for GFAP. **(H)** IHC shows positive staining for EMA.

Macroscopically, the tumor tissue appeared dark gray with a soft consistency. Microscopic examination revealed a diffuse proliferation of neoplastic cells infiltrating the dura and brain parenchyma. These cells exhibited round to oval, polygonal to spindle-shaped morphology, with eosinophilic cytoplasm, a high N/C ratio, oval nuclei, moderate to severe nuclear pleomorphism, irregular nuclear membranes, vesicular chromatin, and prominent nucleoli (Figures 3A–C). Mitotic figures were observed at a rate of 2 per 10 high-power fields. Dense melanin pigmentation was noted among the tumor cells. Immunohistochemical examination was performed on the patient, where the specimen tested positive for Melan-A, human melanoma black 45 (HMB45), S100, glial fibrillary acidic protein (GFAP), and epithelial membrane antigen (EMA) (Figure 3D–H). Based on these histopathological and immunohistochemical findings, the tumor was identified as a primary intracranial malignant melanoma.

### 2.5 Postoperative management

**Figure 4:**
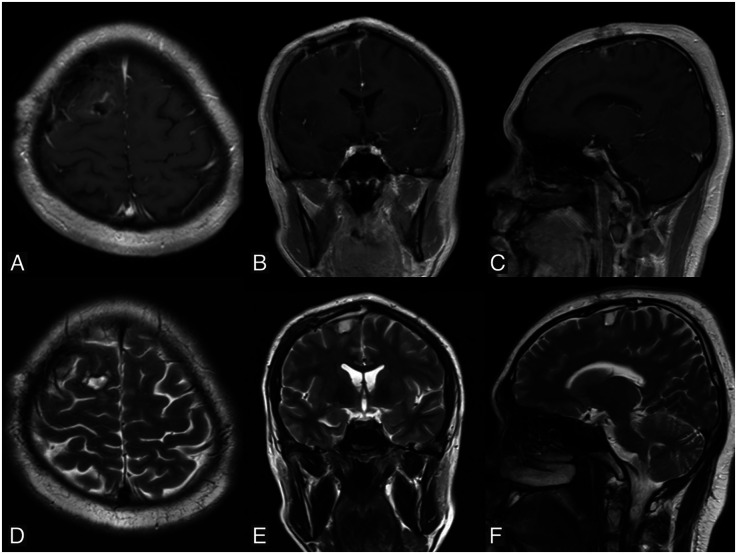
Follow-up MRI performed one month postoperatively reveals a residual mass in the right parasagittal frontal region. **(A–C)** Are T1-weighted images with contrast in axial, coronal, and sagittal views, respectively. (**D–F)** Are T2-weighted images in axial, coronal, and sagittal views, respectively.

The patient was hospitalized for four days, during which no seizures or neurological deficits were observed. A whole-body PET scan showed no evidence of melanoma elsewhere in the body. The patient was scheduled for a follow-up MRI one month later, which revealed a residual mass in the right frontal region (Figures 4A–F). The patient was planned for reoperation and radiotherapy, but declined due to financial reasons. One year after the surgery, the patient remained seizure-free, without any neurological deficits, and was able to resume daily activities without limitations. Unfortunately, the patient continued to refuse a follow-up MRI and further treatment due to financial constraints.

## 3 Discussion

Melanosomic melanin differs from neuromelanin, as it is only found in nerves (4). Leptomeningeal melanocytes originate from the neural crest (5). The highest concentration of melanocytes in the central nervous system (CNS) is found in the pia mater, around the spinal cord up to the upper cervical cord (6, 7). Primary melanocytic tumors of the CNS are very rarely reported and are histologically and clinically distinct from metastatic malignant melanomas originating from the retina or skin (1). These tumors are classified into diffuse melanocytosis, meningeal melanomatosis, malignant melanoma, and benign melanocytoma, with a few other minor variants (8). According to the WHO classification, these tumors are divided into diffuse meningeal melanocytic neoplasms, which include the sub-classification meningeal melanocytosis and meningeal melanomatosis, and circumscribed meningeal melanocytic neoplasms, which include the sub-classification meningeal melanocytoma and meningeal melanoma (9). The prevalence of these primary melanocytic tumors is low, with an estimated incidence of 0.9 per 10 million people worldwide (6). Among these, primary malignant melanoma of the CNS has an even lower incidence, accounting for only 1% of all melanoma cases and 0.07% of all primary CNS tumors, with a specific incidence of 0.005 cases per 100,000 individuals (10–13). The first case of primary intracranial melanoma was described by Ogle in 1899. These tumors are more commonly observed in males, typically between the ages of 40 and 50, and are extremely rare in adolescents (1, 6, 14). Since 2001, only nine cases of primary intracranial malignant melanoma (PIMM) in patients under 20 years old have been reported in the literature (Table 1) (1, 15–22).

**Table 1:** Reported cases of primary intracranial malignant melanoma in an adolescent (M, male; F, female).

	Authors	Year	Age	Sex	Origin	Treatment	Pathology
1	Desai et al. (22)	2001	17 years	F	Cerebello-pontine angle	Resection and radiotherapy	Yes
2	Son et al. (21)	2003	12 years	M	Convexity	Resection and radiotherapy	Yes
3	Chen et al. (20)	2013	16 years	M	Subcortical	Resection and radiotherapy	Yes
4	Balakrishnan et al. (19)	2015	16 years	M	Subcortical	Resection and chemoradiotherapy	Yes
5	Mondal et al. (18)	2016	11 years	F	Infratentorial Convexity	Resection and radiotherapy	Yes
6	Sivaraju et al. (17)	2019	16 years	M	Convexity	Resection and chemoradiotherapy	Yes
7	Otero-Soto et al. (15)	2020	17 years	F	Parasagital	Resection and chemoradiotherapy	Yes
8	Lim et al. (16)	2023	15 years	M	Parafalcine	Resection and chemotherapy	Yes
9	Vishwajeet et al. (2)	2024	17 years	F	Subcortical	Resection	Yes

These tumors can arise from melanocytes located in the leptomeninges at the brain convexities, skull base, posterior fossa, cervical spinal canal, pia mater covering blood vessels, reticular formation in the pons and spinal cord, substantia nigra, and locus coeruleus (14). The most common locations in the CNS are lobar (53.1%), posterior fossa (17.3%), and pineal region (13.6%) (6). Several theories have been proposed to explain the origin of PIMM (10). The first is the mesodermal theory, which suggests that pigment cells originate from the mesoderm and reach the brain or spinal cord through pial blood vessels. The second is the ectodermal theory, which posits that since only epithelial cells produce pigment, PIMM arises from aberrant embryonic ectodermal cells. The third theory is the neurogenic theory, which suggests that pigment cells originate from the neural crest and can lead to tumor growth. Chromosomal abnormalities have also been reported, such as deletions or ruptures of the long and short arms of chromosome 6, resulting in the loss of tumor suppressor gene function in PIMM (23).

Patients with PIMM typically present with various symptoms, which are secondary to the local effects on CNS parenchyma, including increased intracranial pressure and hydrocephalus (43.2%), focal neurological deficits due to mass effect (34.6%), subarachnoid hemorrhage (17.3%), and seizures (11.1%), as observed in our case (1). Symptoms can also appear acutely, usually associated with intratumoral hemorrhage, which is often reported in these tumors (23). Rapid progression with increased intracranial pressure may indicate malignant transformation, leading to irritability, vomiting, lethargy, seizures, and more (13). The differentiation between primary and secondary melanoma lies in that primary CNS melanoma arises intracranially from melanocytes typically located in the leptomeninges or dura, whereas secondary (metastatic) melanoma occurs both intracranially and extracranially, often due to hematogenous spread from a known or unknown primary tumor, with characteristic involvement of the parenchyma or leptomeninges (24). Dermatological screening for primary lesions involves assessing features such as asymmetry, border irregularity, color variegation, a lesion that looks different from others, changes over time (25).

The characteristic imaging features of PIMM on non-contrast CT scans typically show hyperdense lesions (6). On MRI, these tumors present with hyperintense T1 and hypointense T2 signals, due to the paramagnetic effect of stable free radicals within melanin (6, 26). The interaction between the unpaired electrons in melanin and the protons in water produces dipole-dipole interactions that cause hyperintensity on T1 and hypointensity on T2 (4). However, some variations exist, including T1 hypointensity and T2 hyperintensity, usually observed in tumors with less than 10% melanin content in the cells or in cases of intratumoral hemorrhage (11, 26). Leptomeningeal dissemination may also be observed (6). Other intracranial tumors should be considered in the differential diagnosis of PIMM, such as meningioma, medulloblastoma, astrocytoma, melanotic schwannoma, pituitary tumors, and choroid plexus papilloma (6, 13).

Diagnosing melanoma as truly primary to the CNS or a metastasis from another site remains controversial. Hayward proposed a classification for CNS melanomas as follows: (1) primary CNS melanoma, (2) secondary CNS melanoma, and (3) variations of other intracranial tumors with melanin (27). Several factors are necessary for classifying these tumors: (1) absence of melanoma outside the CNS, (2) tumor located in the cranial or spinal leptomeninges, (3) intramedullary spinal lesions, (4) hydrocephalus, (5) tumors in the pineal or pituitary areas, and 6) solitary cerebral lesions (27). These criteria are still relevant and are commonly used today because histopathological and immunohistochemical diagnosis often fails to clearly distinguish between primary and secondary CNS melanomas (28). PIMM can be classified as solitary or diffuse. Solitary PIMM is distinguished from diffuse PIMM by its nodular mass, consistent with its pathological behavior. When diffuse PIMM infiltrates the pia mater and subarachnoid space, it leads to a poor prognosis and subtotal tumor resection, while solitary PIMM may undergo aggressive treatment with a potential for prolonged survival (13).

Definitive diagnosis of CNS melanoma is established through histopathological examination (1). In 70% of cases, these tumors appear dark during intraoperative visualization (5). Common histopathological findings in primary CNS malignant melanoma include pigmented tumor cells, cytological abnormalities, CNS invasion, pleomorphic nuclei, and high cellularity, arranged in syncytial, epithelioid, or irregular conglomerates of pigmented cells with leptomeningeal infiltration (5). However, a poor prognosis is observed when lesions are large, firm, deeply invasive, exhibit an epithelioid cell pattern, and have high mitotic activity (22, 29). Immunohistochemistry can aid in diagnosing melanoma, using markers such as S100 protein, HMB45, and Melan-A (6, 13). These tumors generally test negative for GFAP and EMA (30). However, in some cases, GFAP and EMA may show positive staining, as reported in the literature (3, 31).

The majority of authors concur that there is no established standard treatment, and the effectiveness of radiotherapy and chemotherapy for primary malignant melanoma of the brain remains a topic of debate (32). The primary treatment for this condition is surgical intervention (1, 6). Gross tumor resection is crucial for patient survival. Patients undergoing gross tumor resection have a longer life expectancy (>22 months) or a 40.8% three-year survival rate compared to those who do not undergo surgery (33, 34). Aggressive surgical management, including complete resection and even repeated excision, is key to extending survival regardless of tumor size or location (13). Adjuvant radiotherapy following tumor resection is beneficial for survival in metastatic melanoma and PIMM (33). Recent studies suggest that stereotactic radiosurgery (SRS), as an alternative to whole-brain radiotherapy (WBRT), may be effective when combined with immunotherapy or targeted therapy. However, further prospective studies are needed to confirm the efficacy of these emerging treatment regimens (13). In the present case, we were only able to perform subtotal tumor resection due to the tumor's attachment to eloquent brain areas. The tumor was attached to precentral gyrus which is the primary motor cortex and responsible for controlling voluntary muscle movement at the contralateral side of the body. The patient came without any motoric neurological deficit and more extensive resection would pose a significant risk of new motoric deficits which could be devastating for the patient. We recommended further surgical intervention and radiotherapy to the patient, but they declined due to financial constraints and associated risks.

When tumor recurrence is observed on follow-up MRI, gross total resection remains the first-line treatment, if feasible (13). For diffuse leptomeningeal spread, focal radiation may not be applicable, making SRS an alternative approach. WBRT can be considered if the patient and family understand the potential neurological dysfunction caused by radiation. Terminal stages are marked by hydrocephalus refractory to curative procedures, as seen on imaging. CSF cytology may be useful in detecting diffuse leptomeningeal spread (13, 35). Primary malignant melanoma of the brain has a better prognosis than metastatic melanoma, especially if total resection is achieved, with aggressive multimodal management extending survival beyond the 3–6 months typically seen in secondary brain melanoma to about 20.7 months for solitary primary CNS melanoma patients (5, 23, 36). In a study of 84 cases of primary CNS melanoma, Man et al. concluded that age is a significant prognostic factor, with pediatric patients generally having the poorest outcomes. Median survival in pediatric, adult, and elderly populations is approximately 3, 17, and 16 months, respectively (37).

## 4 Conclusion

Primary intracranial malignant melanoma (PIMM) is an exceptionally rare tumor in the central nervous system, with a particularly low incidence in adolescents. This case report highlights an unusual presentation of PIMM in an 18-year-old male, initially presenting with seizures. Through detailed clinical, radiological, and histopathological evaluations, a definitive diagnosis was established, emphasizing the importance of thorough investigation in such rare cases. Complete surgical resection remains crucial for favorable outcomes, with adjuvant therapies playing a role in managing recurrence. Although PIMM carries a better prognosis than metastatic melanoma when aggressively treated, its rarity necessitates more comprehensive studies to guide diagnosis and optimize therapeutic strategies.

## Data Availability

The original contributions presented in the study are included in the article/Supplementary Material, further inquiries can be directed to the corresponding author/s.
